# Policy options to increase the provision of free school meals in England: a qualitative exploration of the challenges of policy implementation through stakeholder interviews

**DOI:** 10.1017/S1368980026102432

**Published:** 2026-06-25

**Authors:** Catrin Pedder Jones, Bisola Osifowora, Viktorija Kesaite, Irina Pokhilenko, Steven Cummins, Rachel Loopstra, Alexia Sawyer, Amy Yau, Martin White, Emma Frew

**Affiliations:** 1 MRC Epidemiology Unit, School of Clinical Medicine, University of Cambridge, UK; 2 Centre for Economics of Obesity, Department of Applied Health Sciences, University of Birmingham, UK; 3 Population Health Innovation Lab, Department of Public Health, Environments and Society, Faculty of Public Health and Policy, London School of Hygiene & Tropical Medicine, UK; 4 Department of Public Health, Policy and Systems, University of Liverpool, Liverpool, UK

**Keywords:** School meals, food insecurity, policy implementation, school meals policy

## Abstract

**Objective:**

Rising levels of household food insecurity in England, and associated health and well-being impacts for children, have led to calls to expand access to free school meals (FSM). Policymakers have been hesitant to extend provision of FSM due to concerns surrounding acceptability, affordability and implementation challenges. The most effective strategies for expanding FSM are not yet fully understood. This work aims to fill this gap by examining school meal policy from the perspective of multiple stakeholders, to provide actionable recommendations for policies that could expand school meal provision.

**Design:**

A qualitative interview study design was used. The data were analysed using the Framework Method, underpinned by the Context and Implementation of Complex Interventions (CICI) framework. Themes were categorised into context-related and implementation-related factors. Detailed recommendations were discussed at the macro, meso and micro levels of the school food system.

**Setting:**

The study was conducted in England, UK.

**Participants:**

Seventeen stakeholders represented the views of local, regional and national government, policy, academia and schools.

**Results:**

Stakeholders indicated that policies should prioritise stigma reduction and integrate expansion of school meals with existing school policies where possible, including the monitoring of school food standards and ensuring maintenance of school food quality. Stakeholders also suggested improvement to the administrative process and communication with families and recommended a joined-up approach linking interventions with common goals across the whole food system.

**Conclusions:**

Crucially, sufficient financial support is essential for successful implementation.

## Introduction

Nineteen per cent of households with children in the UK experienced food insecurity in 2024, defined as an inability to access consistently available safe and nutritious food in quantities needed for normal human growth and a healthy life^(1,2)^. Among households receiving Universal Credit, 38·7 % reported experiencing food insecurity^(2)^. Food insecurity is associated with adverse health, social and economic outcomes, including increased risk of non-communicable diseases such as obesity and diabetes, poor dietary and nutritional outcomes due to reduction in healthy food purchases and reduced educational attainment^(2–5)^. Food-insecure households are more likely to reduce purchases of healthy foods, such as fruits, vegetables, fish, dairy products and eggs, due to their higher cost^(2)^, and numerous studies have linked food insecurity to poor diets and nutritional status^(6)^. In response to concerns about the high level of food insecurity and its health consequences among children, there have been increasing calls for policy options to expand access to free or affordable school meals (FSM) to address malnutrition and reduce health and social inequalities in England^(7)^. Access to a FSM has been shown to reduce school absence and improve educational attainment leading to longer-term productivity gains^(8)^. However, universal access to FSM is not the current policy in England.

In England, all Key Stage 1 (ages 4–7 years) pupils receive FSM. In Key Stage 2 (ages 7–11 years), FSM are means-tested, with eligibility based on households receiving income-replacement benefits; in 2024, 24·9 of KS2 pupils qualified^(9)^. Although secondary pupils are also assessed through a means-tested system, this study focused on primary school provision^(10)^. In Greater London, universal provision of FSM for primary school pupils has been provided since 2023, an initiative launched by the Mayor of London and funded by business rate income^(11)^. For primary school pupils in the rest of England, households have the option to pay for a school meal, with the average cost in England at £2·70 per meal, or bring in a packed lunch from home^(9)^. While some progress has been made towards improving access to FSM, advocacy groups, politicians, parents and the media are increasing pressure to expand FSM provision owing to increasing levels of food insecurity^(12–20)^.

Several policy options for increasing access to school meals have been suggested such as making FSM universally available to all primary and secondary school pupils^(20,21)^, ensuring automatic enrolment for children eligible for FSM to remove the need for families to opt in^(22)^, expanding provision to all pupils in schools in areas of high deprivation^(14)^ or expanding eligibility to include all pupils from families in receipt of Universal Credit, regardless of income levels^(21)^. Yet despite significant lobbying on this issue, there are few examples of either local or national policymakers implementing these types of policies. However, since completing data collection for this study, the UK government announced that from September 2026, access to FSM in England will be extended to all children from households receiving Universal Credit, alongside a review of the school food standards^(23)^. While this is a positive step, substantial work remains to ensure that every child can fully benefit from a nutritious school lunch, including consideration of more universal provision.

Policymakers’ hesitance in expanding FSM may come from concerns over political acceptability and affordability^(24,25)^. With increased advocacy for FSM, there is an urgent need for high-quality research to guide policy implementation and assess the implications of these policies^(26)^. This will help with the implementation of the new policy coming in September 2026, as well as later advocacy work to expand FSM further.

Previous research has explored the implementation of various school meal policies through the perspectives of school staff, parents and pupils^(27–29)^. Feedback from two London schools taking part in universal FSM found it was easier to implement than the previous ‘opt-in’ policy with thresholds for eligibility due to all pupils receiving a FSM. However, school infrastructure, including dining space, was a barrier to implementation, as well as increased queuing times, lower meal quality and less choice^(28)^. In another study, recommendations on how to increase FSM uptake were developed by consulting school staff and pupils in Leeds and these includes the need for better understanding of factors involved in uptake, a simple registration process that minimises discrimination and maximises awareness, a pupil-centred approach to food quality and understanding the social elements of mealtimes for students^(27)^. Similar implementation issues were identified in the Department for Education FSM pilot evaluation by Kitchen et al. (2013)^(30)^. In Scotland, for universal free school breakfasts and extending FSM, senior policymakers noted the importance of good communication, having flexible timetables, the existence of an already high uptake rate, ensuring popular menu items and taster sessions for pupils as key facilitators, while the barriers included lack of funding, staff recruitment and space constraints^(29,31,32)^.

Collaboration and good communication between all levels of school food provision when developing and implementing health-promoting policies have been recommended more generally^(33)^. However, a comprehensive understanding of the implementation challenges relating to different policy options to expand access to a school meal is lacking. This study aimed to understand potential implementation challenges for national government, local authorities and schools and to generate stakeholder-informed recommendations for implementing policies to expand access to school meals in England. In doing so, the study explored various policy options including provision of FSM, as well as a subsidised policy leading to school meals being more affordable. From here on, the term ‘FSM’ is used to refer both to FSM and to subsidised meals that reduce the cost to families.

## Methods

### Design

Interviews facilitated by a topic guide were conducted with a variety of food and school system stakeholders (described below). Analysis of these interviews adopted a critical realist^(34)^, theoretically guided, deductive approach. The selection of critical realism aligns with theory and researcher-driven deductive approaches to research, prizes the integration of context within analysis and is conducive to the development of policy-related recommendations^(35)^. Critical realism facilitated the development of implementation recommendations through the integration of stakeholder perceptions within a multi-level theoretical framework: the Context and Implementation of Complex Interventions (CICI) framework^(36)^.

### Research team

*[Redacted to facilitate anonymous peer review].* The team were reflexive throughout the interviews and analysis, to examine their influence over the interpretation of the results. For example, team members kept brief reflexive notes after interviews to record their assumptions and reactions, and these were discussed in meetings when interpreting the data. A descriptive approach to the analysis prioritised participants’ views in their own words. Regular informal meetings of the research team took place throughout the analysis, followed by a formal data clinic to incorporate the wide variety of topic expertise in interpreting the findings.

### Data collection

#### Participants and setting

Stakeholders in England were identified from regional government, local government, schools, food policy advocates and academia and were chosen for their expertise across the school food system and in related policy areas. Initial contacts were identified from the [redacted – study teams] networks, followed by snowball sampling via email. Interviews were conducted via Zoom between 19 January and 9 February 2024.

#### Ethics

Ethical approval was provided by the University of Cambridge Humanities and Social Sciences Research Ethics Committee (approval no. 21288).

#### Materials and procedure

During the interviews, the participants were presented with a list of policy options (Table 1) to increase access to school meals, informed by a scoping literature review and expert opinion, a selection criterion (taken from Chapter 4, pp57–75, Caputo 2013^(37)^) (Appendix B, Table B1) and a draft conceptual model (Appendix B, Figure B1) to help structure discussion about the relevant cost implications and outcomes generated from expanding school meals (published elsewhere). Participants felt that the overall logic and components of the model broadly made sense but highlighted key assumptions and potential unintended consequences (such as increased food waste, longer queues and reduced eating time at lunch, and implications for pupil premium funding) that needed to be considered when interpreting the results. A semi-structured interview topic guide and short survey, containing both qualitative and quantitative questions, was used to guide the interviews (Appendix A), and some interviews were conducted with pairs of participants. Some of the quantitative questions asked included participants rating the perceived importance and feasibility of different policy options, time horizons and outputs for model results; these responses were summarised graphically (Appendix C) and used to prioritise and guide decisions in the subsequent modelling work. Interviews were transcribed verbatim and then checked for accuracy against recordings by [Author 1].

**Table 1.** Policy options for the provision of free school meals

Current policy		
**Universal infant free school meals**		Since September 2014, state-funded schools in England have been mandated to provide free lunches to pupils in reception, year 1 and year 2 who do not qualify for benefits-related free school meals (Education & Skills Funding Agency, 2023).
**New policy options to be considered**		
1	Providing FSM **to all children** in primary school	All primary schoolchildren receive a free school lunch.
2	Providing FSM to children in **receipt of Universal Credit**	To qualify for free school meals, the child must live in a household who receives Universal Credit.
3	Widening eligibility by **increasing the earnings threshold**	Widening eligibility to all children with a household net income not exceeding £20 000, for example, increasing the earnings threshold from £7400 to £20 000 (post-tax household income).
4	Providing FSM to children whose parents are in **receipt of (any) income support.** Consider the following options listed	4a: receipt of income-based job seekers’ allowance 4b: in receipt of income-related Employment and Support Allowance 4c: in receipt of support under Part VI of the Immigration and Asylum Act 1999 4d: in receipt of Child Tax Credit
5	Providing FSM to all pupils who **attend 25 % leas**t **advantaged schools**	The selection of schools will be based on a geographic index of deprivation such as the Index of Multiple Deprivation (IMD) score or the IDACI score, using the school postcode.
6	Providing FSM to all pupils who attend schools with **>50 % of pupils currently eligible for FSM**	Identify schools based on current eligibility criteria. All schools with >50 % of pupils currently eligible (as of January 24) for FSM.
7	All parents of children ineligible for a FSM, pay a subsidy towards a school lunch (meaning all pupils receive a school lunch).	Subsidy to be determined by further research.

FSM, free school meals.

### Analysis

#### Theoretical framework

Selection of an analytic theoretical framework was informed by a systematic review which compared food and physical activity policy implementation frameworks^(38)^. The CICI framework^(36)^ was selected for its comprehensiveness, particularly because it incorporates many levels of context aligned with the different stakeholder perspectives (national, regional and school levels)^(38)^. Developed by Pfadenhauer et al. (2017), it aims to integrate the CICI across macro (national), meso (regional/school) and micro (individual) levels. CICI was used as a ‘determinant framework’ to ‘conceptualise, describe and understand the multiple influences on implementation outcomes’ as defined by the framework authors^(36)^. The components within the CICI framework include ‘Context’ (socio-economic, sociocultural, ethical, legal, political, epidemiological, geographical) and ‘Implementation’ (process, strategy, agents and 153 outcomes)^(36)^ and were used to structure the deductive analysis and facilitate the description of implementation recommendations for policymakers choosing to expand FSM across England (Appendix D).

#### Analysis method

Interviews were transcribed and then analysed in NVivo 12^(39)^ using the Framework Analysis method^(40)^. Framework Analysis, a type of thematic analysis, is typically used to explore qualitative data comparatively across cases (participants) with the use of a matrix and was developed specifically for policy-based research^(41,42)^. Framework adopts the following stages: (i) transcription, (ii) familiarisation, (iii) coding, (iv) developing a working analytical framework, (v) applying the analytical framework, (vi) charting the data into the framework matrix (in this case, a matrix was used to explore participant responses across sectors according to the CICI framework) and (vii) interpretation.

Familiarisation began with [Author 1] reading the transcripts multiple times, noting initial impressions. The CICI framework was then imported into NVivo, and coding was conducted based on this framework and the definitions provided by the authors of the CICI framework (Appendix D)^(36)^. Data were then charted into a matrix, where the CICI framework and sub-themes (columns) were summarised for each stakeholder group (rows) for the purpose of data reduction. Implementation recommendations for the macro, meso and micro levels were then extracted by synthesising the themes from the context and implementation domains using the CICI framework. All recommendations were descriptively based using the participant responses and are framed throughout as ‘stakeholder recommendations’. Sub-themes within the CICI categories and recommendations were explored and refined further during a data clinic with team members who had conducted the interviews and were familiar with the transcripts (redacted). Finally, [Author 1] drafted the results, and interpretation was further refined with input from all authors.

## Results

Eleven interviews were conducted with seventeen participants, including some paired interviews: one stakeholder from regional government, three food policy advocates, three from local government, four academics and six from a primary school leadership setting. Each lasting about 60 min. Recruitment continued until stakeholders from each targeted part of the food system had been included, and no substantive new issues relevant to our research aim were emerging. Findings are presented according to macro, meso and micro levels, drawing on the CICI framework to show how contextual factors and implementation processes shape the feasibility, acceptability and delivery of policies to expand access to FSM. (*Please see* Appendix E *for full theme details*).

### Context and Implementation of Complex Interventions framework themes

#### Context

##### Macro: government and national level

At the macro level, stakeholders highlighted a range of socio-economic, sociocultural, ethical and political conditions that shaped whether expanding access to FSM was viewed as necessary, affordable and administratively feasible. Socio-economic pressures, particularly the cost-of-living crisis, the complexity of the benefits system, the link between FSM registration and pupil premium funding and transitional protection inflating FSM statistics, increase the urgency to act on child food insecurity while also heightening concerns about fiscal and administrative consequences. Sociocultural debates about the ‘nanny state’ and the appropriate role of government in food provision sit alongside recognition of the positive role of school meals in food culture and food literacy. Ethically, participants framed expansion as a question of fairness and moral obligation, noting that current policies favour some age groups and exclude many low-income working families, thereby strengthening the case for further expansion. Politically, growing advocacy for universal FSM and related campaigns, combined with the constraints of electoral cycles and forthcoming changes to benefits administration, shaped perceptions of how willing and able national policymakers were to commit to substantial policy change.

### Meso: local authority and school level

At the meso level, stakeholders described socio-economic, legal/ethical, political, epidemiological and geographical conditions that shape how any expansion of FSM would be operationalised by local authorities and schools. Socio-economic factors including the financial position of local authorities and schools, the potential for both savings and losses from economies of scale, variation in existing catering arrangements and the use of cost–benefit analyses influence whether schools are seen as able to increase uptake without undermining other priorities. Legal and ethical considerations around schools’ discretion to award FSM under the Education Act^(43)^ create scope to ‘fill gaps’ locally but also introduce variation and uncertainty about how consistently expanded policies would be applied. Political priorities and the presence of a school food ‘champion’ at a local level affect whether authorities are likely to drive, adapt or resist policy changes, and whether they would take on administrative roles such as enrolling pupils. Epidemiological framing of FSM eligibility as a marker of wider family need highlights opportunities to link school meals with broader support but also implies additional coordination and workload. Finally, geographical fragmentation of school structures and catering contracts, and the practical logistics of meal delivery and dining space, constrain how quickly and uniformly policy changes could be implemented across different areas.

### Micro: pupils, parents and teachers level

At the micro level, stakeholders described contextual conditions that shape how policies to expand FSM are experienced by families, pupils and teachers, and whether they translate into greater uptake. Socio-economic pressures on families just below and above current eligibility thresholds mean that even small charges can act as a barrier, affecting whether parents are able to take up school meals for their children. Sociocultural factors including the importance of choice and autonomy, the way packed lunches and school meals are perceived by peers and teachers’ experiences of managing pupils who are hungry or in debt influence the social acceptability of different policy options. Ethical concerns about preserving liberty and avoiding food waste, alongside recognition that FSM eligibility often signals wider family need, highlight opportunities to link school meals with broader support but also raise questions about the appropriate extent of school involvement. Epidemiological framing of the cost-of-living crisis as part of a wider burden on families shows that, without explicit attention to stigma and everyday school practices, expanding eligibility alone may not deliver the intended improvements in diet, well-being and learning.

### Implementation

#### Macro: government and national level

At the macro level, stakeholders emphasised that implementation depends first on how policies are framed and advocated for nationally. Advocacy that centres the lived experiences of children and families benefiting from FSM and clearly communicates the fairness and wider social returns of expansion was viewed as essential for securing political and public support, particularly when FSM compete with other education priorities for resources. Participants suggested that national implementation strategies should operate across all levels of the system and demonstrate impact beyond food alone, for example, by linking FSM expansion to improvements in learning, health and future productivity. To make delivery feasible, stakeholders highlighted the need for clear national guidance and robust administrative and digital systems, including auto-enrolment mechanisms, adjustments to the benefits system and reconsideration of the link between FSM registration and pupil premium funding. Central government, ministers, civil servants, academics and advocacy organisations were all identified as key macro-level implementation agents, and stakeholders argued that monitoring should track not only immediate outcomes (such as registrations and diet quality) but also longer-term feedback loops in education, health and inequalities, which can reinforce or undermine support for policy continuation and scaling.

#### Micro: pupils, parents and teachers level

At the micro level, stakeholders emphasised that successful implementation depends on how policies are experienced in schools and whether they feel acceptable and stigma-free for families, pupils and staff. Across interviews, stigma emerged as a central mechanism: policies that leave some children visibly ‘different’ at mealtimes (e.g. requiring separate registration or parental subsidy) were viewed as unlikely to achieve high uptake among those most in need compared to a universal offer. Participants argued that implementation strategies should therefore explicitly address the social dynamics of lunchtimes, including peer perceptions, parental preferences and the impact on teachers who currently manage hunger, meal debt and distress. Practical ideas included using familiar administrative systems to reduce bureaucracy for families and regularly involving parents and pupils in menu design within the constraints of school food standards, both to support acceptability and to reduce food waste. Families, pupils, parents and teachers were identified as key micro-level implementation agents, and outcomes at this level such as stigma, social relationships, mental health, diet quality, academic engagement and family hardship were seen as critical indicators of whether expanded policies are working as intended.

## Synthesis of multi-level influences

**Figure 1.**
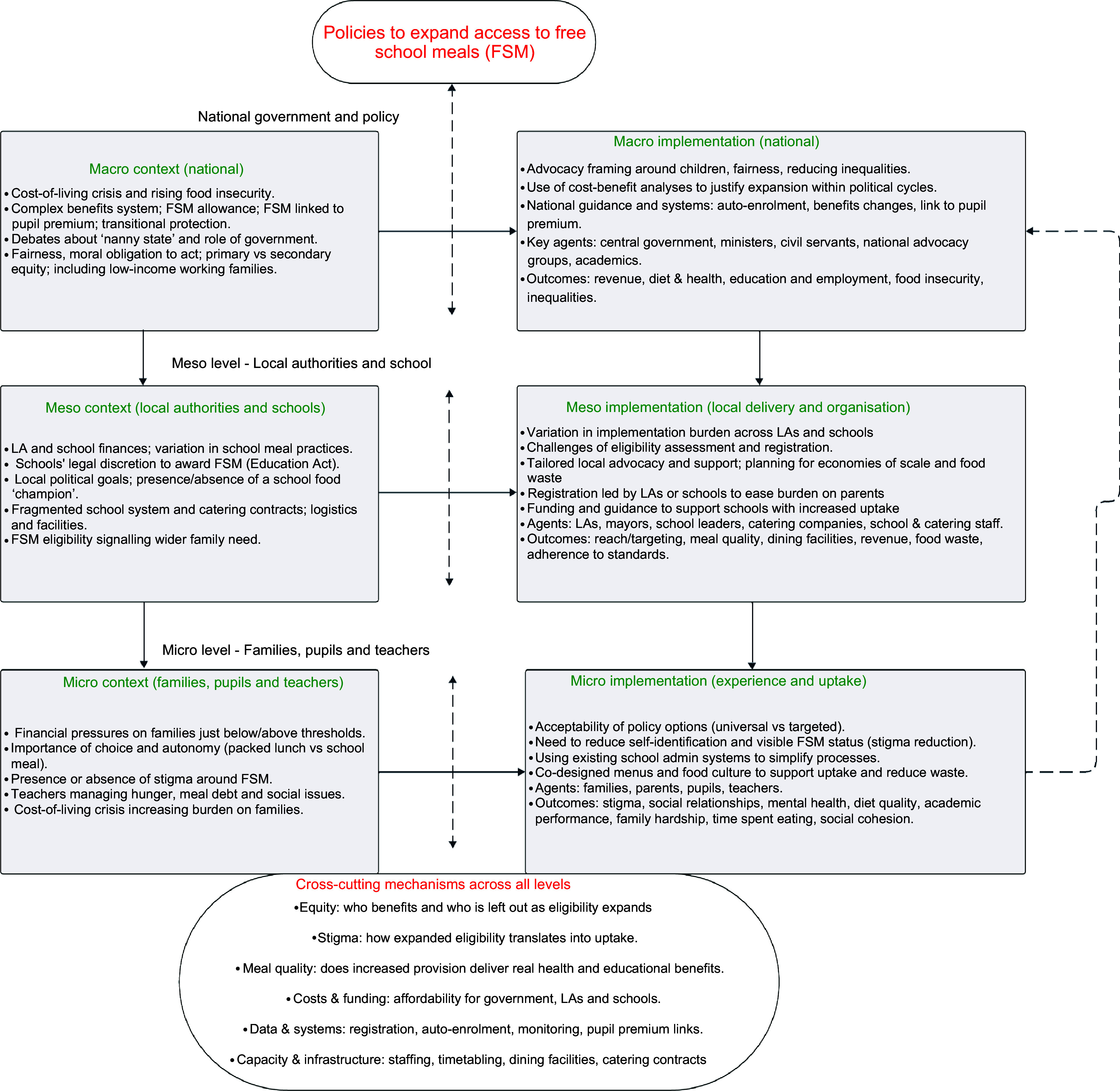
Contextual and implementation influences on policies to expand access to FSM in England, by system level. Left → right within each row = context influencing implementation, top → middle → bottom = macro → meso → micro influence, and curved arrow from micro implementation to macro implementation = feedback/learning from practice. FSM, free school meals.

Taken together, the macro, meso and micro findings show that policies to expand access to FSM are shaped by interacting contextual and implementation factors across the school food system. Figure 1 summarises these multi-level influences, organised using the CICI framework, and highlights cross-cutting mechanisms that inform the implementation recommendations set out below.

## Implementation recommendations for policy to expand school meals

Recommendations for implementing policy changes related to school meals can be viewed in Box 1. The context and implementation themes derived from the CICI framework were further synthesised to identify key recommendations for implementation at the macro, meso and micro levels.

Box 1. Stakeholder Recommendations for Implementing School Meal Policies

**Government-level (macro) implementation**

1. **Consider existing school food policies for low-income families:**
  - *Understand how current policies, particularly their administration, interact with any new policy being implemented. For example, the UK’s transitional protection policy ensures that pupils registered for FSM retain their entitlement even if their family becomes ineligible. This can lead to discrepancies in FSM statistics, making it crucial to accurately reflect ‘real’ eligibility.*
2. **Examine the link between low-income benefit registration and school meal policies:**
  - *The complexity of national benefits systems can impact delivery of school meal policies. Local authorities may face challenges in accessing benefit data to facilitate enrolment.*
3. **Prepare arguments for opponents of new policies:**
  - *Anticipate counterarguments from opponents regarding new policies. Preparing responses to such pushback can enhance the acceptability of policy changes on a national scale, particularly concerns about prioritising food spending over other educational areas.*
4. **Implement policies that preserve autonomy and reduce stigma:**
  - *Some policies can inadvertently increase stigma for pupils and families receiving school meal support. For example, a universal offer eliminates the identification of students needing FSM, whereas a parent subsidy option might lead to feelings of paying for others’ meals, thereby increasing stigma.*
5. **Prioritise monitoring of adherence to existing school food standards**
  - *National monitoring of whether schools comply with current school food standards and related policies is limited. Governments should strengthen systems for assessing compliance with these existing standards and previously implemented policies, to ensure pupils receive healthy and nutritious food.*
6. **Address financial constraints on regional/local authorities and schools:**
  - *Appropriate financial resources must be allocated from the national level to local authorities as well as to schools to support the implementation of new policies.*
7. **Consider one-off capital cost for school facilities:**
  - *The variability of catering and dining facilities among schools necessitates that central government accounts for the initial capital required to upgrade these facilities in response to increased meal uptake.*

**Regional/local authority (meso)-level implementation**

1. **Account for different school structures in policy implementation:**
  - *Recognise that schools vary in structure, such as primary, secondary and middle schools, as well as academies. Local authorities must tailor FSM policies to accommodate these differences.*
2. **Ensure local authority access to necessary data:**
  - *Since government-level benefits data (like Universal Credit) is often not provided to local authorities, they should establish systems to collect this data either from parents or schools directly, or the government where necessary*.
3. **Communication changes in FSM registration processes effectively:**
  - *Local authorities must ensure schools and parents are informed about any variations in registration processes, such as who is responsible for registering to facilitate proper policy implementation.*
4. **Minimise registration burden on families to reduce stigma:**
  - *Any policy, except universal free school meals, should aim to minimise the burden on pupils and families. Auto-enrolment is preferred, as stigma may deter parents from registering for FSM.*
5. **Strengthen local monitoring of school food standards:**
  - *Local authorities should strengthen monitoring of compliance with school food standards in the schools under their remit, complementing national monitoring arrangements, to maximise the positive benefits of increased school meal uptake.*
6. **Provide wider support for families registered for FSM:**
  - *Eligibility for FSM should signal to local and regional governments that families may require more assistance beyond food. Systems should be implemented to link this eligibility with other available benefits.*
7. **Link policy implementation to other interventions:**
  - *School meals policies can be integrated with other food-related initiatives to improve implementation and create opportunities for systemic changes within local authorities and regional governments. For example, linking FSM policy with interventions aimed at reducing or monitoring food quality standards.*

**School (micro) implementation recommendations**

1. **Adapt staffing and timetabling to manage increased school meals uptake:**
  - *Schools and catering providers should work together to ensure sufficient kitchen staff and lunchtime supervision. Where catering is contracted out, providers should plan adequate catering staff capacity, while schools may need to adjust timetables and dining-room supervision to accommodate more pupils eating at school.*
2. **Limit financial burden on families and teachers:**
  - *Strategies should be developed to mitigate the accumulation and impact of school meal-related debt for ineligible students. For example, systems to remind pupils regularly of debt to ensure large debts are avoided or to offer different payment options.*
3. **Encourage appointment of a school food champion:**
  - *Schools should appoint a staff member as a school food champion, dedicated to engaging with pupils, parents and staff on menu planning, and fostering a positive food culture.*
4. **Foster a positive school food culture alongside other interventions:**
  - *Implementing school meal policies presents an opportunity for educational interventions as well as promoting positive social interaction around food.*
5. **Integrate stigma prevention into school meal processes:**
  - *All policies, except universal FSM, should allow parents and pupils to avoid self-identification as needing FSM. Measures should be taken to ensure discretion during mealtimes to prevent stigma, allowing pupils on FSM the same choices as those paying*.
6. **Utilise economies of scale to enhance benefits from increased uptake:**
  - *Schools should capitalise on the economies of scale that can arise as a result of increased uptake.*
7. **Integrate discretionary policies to avoid ‘cliff edge’ effects:**
  - *Schools should be aware they have the discretion to award FSM to any pupil they deem necessary, as outlined in the Education Act. While funding may not be provided for this, it enables schools to support pupils in need.*

## Discussion

### Summary

The aim of this research was to better understand potential implementation challenges for the government, local authorities and schools related to policies that have the potential to increase access to school meals in England. Through engagement with a wide range of professionals with expertise in the school food system, key contextual factors were identified and translated into implementation recommendations for FSM policy. The CICI framework was used to analyse these interviews across the macro, meso and micro levels according to the contextual and implementation-related themes. Policymakers considering changes to school food policy should consider these actions. Stakeholder recommendations for policies that have the potential to increase access to FSM suggest the importance of linking with other existing policies to support low-income families so as to avoid increasing stigma, the regular assessment of school food quality, and that schools are appropriately financially supported to cope with an increase in provision. Stakeholders also suggested improving the collection and management of, and access to, data to support communication with families, and adherence to school food standards. Finally, it was recommended that action be taken to develop a joined-up approach which links interventions across the whole food system with common goals.

### Strengths and limitations

Strengths of this work are that it is the first study to examine a multi-level stakeholder perspective of school food policy implementation in England. The inclusion of a wide variety of stakeholders involved across different parts of the school food system, including schools, regional, local and national policymakers, as well as academics has allowed for stakeholder recommendations to be elicited across different levels (national, regional/local and school). Our work also presented participants with a list of different policy options including universal FSM, increasing the current thresholds for eligibility, changing the method of enrolment, using ‘entire school’-based selection criteria as opposed to pupil-specific criteria and a subsidised policy. Participants were presented with a draft conceptual model to help structure the discussion on likely costs and benefits associated with different policy options. This model and policy options provided important prompts for discussion on the wider school food system. Therefore, findings and recommendations from stakeholders can be applied more widely than for a single FSM policy, for example, only universal FSM, and are based on discussions inclusive of the wider school food system. Using the CICI framework was a strength in that it supported a systematic, multi-level description of contextual and implementation factors surrounding policies to expand school meals. However, CICI organises these influences into domains rather than explicitly representing the dynamic relationships and feedback loops between them. Discussions concerned hypothetical policy options and a draft conceptual model based on an academic literature review. Therefore, our findings may not include some policy-specific elements important for implementation that would only become apparent following actual policy change. A further potential limitation is that the lead analyst did not conduct the interviews; therefore, body language, visual non-verbal cues and interactions are not captured in the analysis. In order to prevent the influence of this, a descriptive approach was selected to keep findings as close to participants’ words as possible. Additionally, the implementation recommendations in Box 1 were synthesised by the research team and were not co-produced or formally validated with stakeholders.

### Relationship to prior knowledge

Our findings support previous work on the implementation of school meal policies: recommendations of auto-enrolment^(22,27,31,32)^, the preservation of parent and pupil autonomy^(44)^, fairness^(28,45)^, monitoring adherence to food standards and other intervention^(22,31)^, increased funding for schools^(28,31,32,44,45)^, planning^(31)^, communication^(32,44,45)^, linking policies to others^(31,44)^, encouragement and preservation of school food culture^(27,31,31,44,45)^ and school staff buy-in^(45)^. Building on this work, we include recommendations specific to different levels of implementation, as previous work has been dominated by a focus on school-level implementation and granularity at the pupil and parent level. Integrating implementation recommendations made by stakeholders at a national, regional and school level may enable more comprehensive implementation across the food system. In addition, the predominant focus of previous work on school catering staff, pupils, parents and implementation by schools is complemented by our work across multiple levels of the food and political systems.

### Interpretation and implications for policymakers

In October 2024, the House of Lords (HoL) Committee on Food, Diet and Obesity produced a report containing recommendations for government to address the ‘Broken Food System’, with FSM identified as representing ‘a crucial opportunity to improve the diets of children in the poorest families’^(46)^. The UK government is urged in this report to review extending free or subsidised provision of FSM and remove technical barriers to accessing FSM via auto-enrolment. The stakeholder recommendations resulting from our work can be used by advocates and politicians at a national level, in response to these calls from the HoL, to support implementation of new school meal policies practically. The UK government’s recent announcement on the expansion of FSM to all children in households receiving Universal Credit, alongside a review of the school food standards, closely aligns with stakeholders’ calls to expand eligibility for FSM and to strengthen monitoring of meal quality and standards. However, our findings indicate that expansion alone will not be sufficient; sustainable funding, adequate infrastructure and staffing and clear implementation support will still be needed to ensure that increased access translates into high-quality meals in practice. Our work can also be integrated into national policy advocacy, particularly following the publication in November 2024 of a report focused on how to encourage food system transformation at a parliamentary level^(47)^. Interviews with former prime ministers, a deputy prime minister, health secretaries and other politicians resulted in the development of ‘ingredients for success’ in the implementation of successful food policy^(47)^. The importance of deploying a compelling argument by focusing on children, emphasising fairness and giving the gift of hope^(47)^ was recommended, which our findings can facilitate. Our findings support emphasising fairness through stigma reduction in school food and help provide schools and local authorities with practical solutions for policy implementation, which could assure politicians and decision-makers of their effective delivery.

At the local and school levels, our findings can help policymakers and implementation agents understand potential operational challenges prior to policy implementation. Public sector institutions, such as schools, are not set up with their primary function to be the provision of food. Multiple complex systems interact and operate within each other, with school food systems operating with local government food systems and national food systems. Therefore, understanding the operational challenges across these sub-systems can help guide migration towards new policies and facilitate successful school food policy implementation.

### Unanswered questions and future research

With the momentum in the UK towards extending and improving access to FSM, it is important to explore and evaluate how any future selection of these policies occurs, as well as to assess their implementation and monitoring adherence. Previous research found a lack of monitoring of obesity policy^(48)^, and the HoL report also recommended monitoring of school food standards^(46)^. Alongside monitoring of school food standards, compliance with any future school meals policy should also be assessed. In addition, further research could investigate school food policy implementation in practice when/if changes are made and compare these to the findings reported in this paper. Future research could build on this work by combining frameworks such as CICI with explicit systems approaches co-developed with stakeholders, to better capture dynamic interactions and feedback loops between national policy, local delivery systems and families’ experiences.

## Supporting information

10.1017/S1368980026102432.sm001 Jones et al. supplementary material Jones et al. supplementary material

## Supplementary material

For supplementary material accompanying this paper visit https://doi.org/10.1017/S1368980026102432
