# Supplementary material for: Policy options to increase the provision of free school meals in England: a qualitative exploration of the challenges of policy implementation through stakeholder interviews
Source: Public Health Nutr. 2026 Jun 25;29(1):e129. doi: 10.1017/S1368980026102432 (PMC13434569; doi:10.1017/S1368980026102432)
Supplement: Supplementary file 1 [file S1368980026102432sup001.docx]

**Appendix A: Interview Topic Guide**

I. Study ID:
II. Which group best describes your role?

- Regional government
- Local government
- Schools
- Academic
- Member of the public
- Policy

*When considering the various policy options for increasing eligibility for school lunches, we would like you to consider the following factors (Document 1: share policy criteria with participant)*

*Now we would like you to look at the list of policy options that we have created (Document 2 share policy options with participants).*

III. First, can you tell us if there are any policy options missing that you feel are important to consider?
IV. Assessment of policy options for inclusion of the model policy importance and feasibility

1. Providing FSMs to all children in primary school

- Very important (must be in model)
- Important (ideally in model)
- Slightly important (if room to include in model)
- Not important
- Not feasible to identify children

2. Providing FSMs to children in receipt of Universal Credit

- Very important (must be in model)
- Important (ideally in model)
- Slightly important (if room to include in model)
- Not important
- Not feasible to identify children

3. Widening eligibility by increasing the earning threshold

- Very important (must be in model)
- Important (ideally in model)
- Slightly important (if room to include in model)
- Not important
- Not feasible to identify children

4a. School level characteristics: 25% least advantaged schools (IMD or IDACI)

- Very important (must be in model)
- Important (ideally in model)
- Slightly important (if room to include in model)
- Not important
- Not feasible to identify children

4b. School level characteristics: schools with >50% pupils currently eligible for FSM.

- Very important (must be in model)
- Important (ideally in model)
- Slightly important (if room to include in model)
- Not important
- Not feasible to identify children

5. Parents pay a subsidy towards a school lunch

- Very important (must be in model)
- Important (ideally in model)
- Slightly important (if room to include in model)
- Not important
- Not feasible to identify children

V. Please can you explain your reasons for your response to QIV

*Now please look at our conceptual model (Document 3) developed to track the relationship between increasing the uptake of a school lunch and the outcomes (either intended or unintended).*

VI. Please take a few minutes to consider the model and all the various steps. Do all the steps within the model make sense?
VII. If no, please tell us why the steps do not make sense?
VIII. Are there any steps missing within the model?
IX. If yes, please tell us what steps are missing:
X. The model can generate different types of evidence (see below). What outputs would be most useful to you ?

*Financial cost benefit analysis - focuses on financial aspect, evaluating the net benefit of an investment by comparing costs and benefits to the taxpayer. It emphasises quantifying and comparing financial transactions or cash flows within a specified time frame.*

- *very useful*
- *somewhat useful*
- *not useful at all*

*Economic cost benefit analysis - adopts a more comprehensive approach, covering not just financial aspects but also broader economic and societal factors. The goal is to measure impact on entire economy by comparing both monetary and non-monetary costs and benefits.*

- *very useful*
- *somewhat useful*
- *not useful at all*

*Local authority cost benefit analysis*

- *very useful*
- *somewhat useful*
- *not useful at all*

*Changes in costs to: Government agencies (Dept of Education); Schools; Parents/Guardians; Catering services/food suppliers; Local businesses; Local authorities*

- *very useful*
- *somewhat useful*
- *not useful at all*

*Changes in outcomes to: Government agencies; Schools; Parents/Guardians; Catering services/food suppliers; Local businesses; Local authorities*

- *very useful*
- *somewhat useful*
- *not useful at all*

11. What outcomes are most important to you?

*HEALTH AND NUTRITION, for example: - increased uptake of school meals; improved diet due to increase in fruit & veg; NHS cost savings due to reduced diet related disease*

- *Very Important*
- *Important*
- *Slightly important*
- *Not important*

*EDUCATION AND EMPLOYMENT, for example: improved educational attainment; increase in lifetime earnings; increase cost savings to schools*

- *Very Important*
- *Important*
- *Slightly important*
- *Not important*

*SCHOOL FOOD AND ECONOMY, for example: improvements to local economy due to increased spending; increased savings on food costs for families due to reduced household expenditures on paid meals and school lunches*

- *Very Important*
- *Important*
- *Slightly important*
- *Not important*

*ENVIRONMENTAL, for example: reduced food waste*

- *Very Important*
- *Important*
- *Slightly important*
- *Not important*

*SOCIAL CONNECTIONS, for example: increased sense of community, enhanced social skills, peer relationships*

- *Very Important*
- *Important*
- *Slightly important*
- *Not important*

XI. Over what time frame would you be most interested in the results being presented (please select)?
    a. 1 year
    b. 5 years
    c. 10 years
    d. 20 years
    e. 50 years
    f. Lifetime
XII. Considering the conceptual model, does the logic (theory of how outputs link to outcomes) of the different policy options look logical to you?
XIII. If no, then how can the logic be improved?
XIV. Is it important to have a measure of the uncertainty in the main results presented?
XV. If yes, how do you prefer uncertainty to be communicated?
    g. 95% confidence intervals
    h. best and worst case scenarios
    i. range of possible scenario outcomes under different assumption
    j. other (please specify)
XVI. If other (please specify):
XVII. Is this model, or its results, something you would potentially use?
XVIII. If yes, please tell us what it would be useful for....
XIX. If no, what could be done to make it more useful for you?
XX. Would you be happy to participate in a workshop end of April/beginning of May to reflect on the results from the model?
XXI. Any other comments?

**Appendix B**

**Table B1: DOCUMENT 1**

Important factors to consider when thinking about alternative policies to increase eligibility for school lunches

| Criteria | Description |
| --- | --- |
| Effectiveness | Likelihood of achieving policy goals. |
| Efficiency | Achieving goals in relation to costs |
| Equity | Achieving re-distribution of resources to ensure equity (not equality) in access |
| Liberty | Extent to which policy extends or restricts privacy and individual rights or choices |
| Political feasibility | Likelihood that a policy would be adopted |
| Social acceptability | Extent to which public will accept and support a policy |
| Administrative feasibility | Likelihood that schools or LAs can implement policy, dependant on capacity of school. Likely to favour policy that is simple rather than complicated. |
| Technical feasibility | Consider need for and availability/reliability of technology needed for policy implementation. |
| Stigma | Think about how means tested benefits that are public (or visible) carry negative or stigmatising connotations. |

* https://www.sagepub.com/sites/default/files/upm-assets/58352_book_item_58352.pdf

**Figure B1: Document 2**

**Conceptual Model**

**Appendix C: Quantitative Analysis of Online Survey**

**Question 2:** What group best describes your role?

**Question 4**: Assessment of policy options for inclusion in the model (perception of importance and feasibility)

**4.1: Providing FSM to all children in primary school (universal access)**

**4.2: Providing FSM to children in receipt of Universal Credit**

**4.3: Widening eligibility by increasing the earning threshold**

**4.4a: School level characteristics: 25% least advantaged schools (IMD or IDACI)**

**4.4b: School level characteristics: schools with >50% of pupils currently eligible for FSM**

**4.5: Parents pay a contribution towards a school lunch**

**Question 6: Please take a few minutes to consider the model and all the various steps. Do all the steps within the model make sense?**

**Question 7: If no, please tell us why the steps do not make sense?**

- Need reduction in food insecurity as a short-term outcome.
- Relies on key assumption that quality of the school meal will result in health and nutritious outcomes.
- Output can lead to either an increase or reduction in food waste (More inclined to see an increase in food waste as opposed to a reduction).
- Reduction in catering revenue as a result of increased eligibility of FSMs
- Broader benefits of nutrition, not just NHS savings.
- Behavioural and teacher engagement
- Eating habits due to increased exposure to healthy meals.

**Question 8: Are there any steps missing within the model**

**Question 9: if no?**

- The inclusion of reduced stigma may serve as a short-term social outcome; however, it may not be applicable to all proposed policy options.
- Each overall outcome should be accompanied by a clear title.
- Negative unintended consequences should be considered, such as the potential for increased take-up of school meals leading to longer queues during lunchtime, resulting in shorter time for children to complete their meals. Other considerations include impacts on pupil premium funding.
- Alternative funding mechanisms, such as pupil premium or additional vouchers should be included.

**Question 10: The model can generate different types of evidence (see below). What outputs would be most useful to you?**

**Question 11: What outcomes are most important to you?**

**11.1: HEALTH AND NUTRITION, for example: - increased uptake of school meals; improved diet due to increase in fruit & veg; NHS cost savings due to reduced diet related disease.**

**11.2: EDUCATION AND EMPLOYMENT, for example: improved educational attainment; increase in lifetime earnings; increase cost savings to schools.**

**11.3: SCHOOL FOOD AND ECONOMY, for example: improvements to local economy due to increased spending; increased savings on food costs for families due to reduced household expenditures on paid meals and school lunches.**

**11.4: ENVIRONMENTAL, for example: reduced food waste**

**11.5: SOCIAL CONNECTIONS, for example: increased sense of community, enhanced social skills, peer relationships.**

**Question 12: Over what time frame would you be most interested in the results being presented (please select)?**

**Question 13: Considering the conceptual model, does the logic (theory of how outputs link to outcomes) of the different policy options look logical to you?**

**Question 14: If no?**

- Increased take-up of free school meals does not necessarily lead to increased usage of dining areas, as these spaces are already allocated for various activities and are often overutilised. The emphasis is more on shared meals rather than focusing solely on dining activities.
- It's important to acknowledge that a significant number of children may opt out of school meals due to personal taste preferences.
- Reduced household expenditure should be included as a short-term outcome.

**Question 15: Is it important to have a measure of the uncertainty in the main results presented?**

**Question 16: If yes, how do you prefer uncertainty to be communicated?**

**Question 18. Is this model, or its results, something you would potentially use?**

**Question 19: If yes, please tell us what it would be useful for…**

- Serving as a robust evidence base to effectively influence policymakers towards making informed decisions.
- Advocating and campaigning to policymakers regarding the imperative for change originating from the central government, as well as engaging with local authorities and academic trusts, especially concerning subsidization.
- Advocating for the expansion of Free School Meals (FSM) programs.
- Establishing and fostering engagement with policymakers at both national and local levels

**Question 21: Would you be happy to participate in a workshop end of April/beginning of May to reflect on the results from the model.**

**Appendix D: CICI Framework components forming the deductive analysis framework**

| **CICI Framework Element** | **Element description taken from Pfadenhauer et al. (2017)**^40^ |
| --- | --- |
| Socio-economic context | “This domain comprises that economic resources of a community and the access of a population to these resources. It also shows the relationship between an economy and its society.” |
| Socio-cultural context | “This domain comprises explicit and implicit behaviour patterns, including their embodiment in symbols and artefacts; the essential core of culture consists of historically derived and selected ideas and values that are shared among members of a group. It not only refers to the conditions in which people are born, grow, live, work and age but also embraces the social roles a human being takes on as a family member, community member or citizen and the relationships inherent to these roles. Constructs such as knowledge, beliefs, conceptions, customs, institutions and any other capabilities and habits acquired by a group are covered by this domain.” |
| Ethical context | “The ethical domain embraces reflections of morality, which encompasses beliefs, standards of conduct and principles that guide the behaviour of individuals and institutions. Ethical issues at stake or in conflict, within systematic reviews/health technology assessments of a complex intervention, are addressed.” |
| Legal context | “The legal domain is concerned with the rules and regulations that have been established to protect a population‘s rights and societal interests.” |
| Political context | “The political domain focuses on the distribution of power, assets and interests within a population, as well as the range of organisations involved, their interests and the formal and informal rules that govern interactions between them. The domain also comprises the health care system and the health care system and the securing of its accessibility.” |
| Epidemiological context | “This domain refers to the distribution of disease/conditions, the attributable burden of disease as well as determinants of needs in human populations. Therefore, it also includes demographics.” |
| Geographical context | “The geographical characteristics refer to the broader physical environment, landscapes and resources, both natural and transformed by humans, available at a given location. As such it also comprises the infrastructure at a given location, which could result in geographical isolation.” |
| Implementation process | “The implementation process refers to the social processes, through which interventions are operationalized in an organization or community.” |
| Implementation strategy | “Implementation strategies encompass all methods and means to ensure the adoption and sustainment of interventions. They comprise a set of activities that are chosen and tailored to fit a specific context or to create such a context.” |
| Implementation agents | “Implementation agents comprise all individuals and organisations engaged with (i) deciding to implement a given intervention (e.g. funders, administrators), (ii) implementing this intervention (e.g. providers, advocates, physicians, nurses) or (iii) being the target or otherwise affected by an intervention (e.g. patients and their families, consumers).” |
| Implementation outcomes | “An implementation outcome is the result or implication of the implementation effort and forms part of good monitoring and evaluation practices.” |

**Appendix E: Themes and subthemes mapped onto the CICI Framework**

|  | **Macro** | **Meso** | **Micro** |
| --- | --- | --- | --- |
| **Socio-economic context** | **Cost of living crisis:** The UK is experiencing a cost-of-living crisis which impacts not only food poverty but wider deprivation and associated outcomes.  **Complexity and inadequacy of benefits system:** The current benefits system is not fit for purpose, it is too complex to navigate, the current FSM allowance is not enough for schools, and FSM registration is used to access pupil premium funding.  **Transitional protection:** Pupils registered for FSM will continue to receive them even if their eligibility changes until 2025. Impact statistics on FSM rates are rising, not a real picture due to this.  ***Macro & Meso:***  **Cost benefit analysis key for decision making:** cost benefit analysis is used to make policy decisions by considering both short and long term outcomes. | **Economic impact of increased uptake on schools:** Increased uptake may require financial investment for some schools depending on their catering facilities and may increase surplus, but it can also increase job creation and increase economies of scale.  **Local authorities and schools financially struggling:** Birmingham City Council’s (BCC) bankruptcy, along with the challenging financial position of other local authorities and schools, highlights the need for government funding to support r any policy needs. Schools are also generating less income from catering.  **Variation between local authorities and schools in school meals practices:** Variation across how FSM eligibility is assessed (LA or parents handling it), also schools differ in how much they charge for meals and how FSM allowances are allocated between food and other expenses. There is also variation in the acceptability of transitional protection, school structures, particularly between middle schools and academies. | **Financial challenges on parents:** The working poor who are not eligible for benefits, face rising school meal costs, making it increasingly difficult for parents to afford them. In some Las, pupils are accruing debt with schools and parents is are required to register themselves for FSM. |
| **Socio-cultural context** | **Positives of school food on food culture**: School food can increase food literacy and bring social connection through it being ‘right’ to provide FSM.  **Current provision not good enough:** There was a shared sentiment that the current provision is not good enough, Many expressed that the UK is experiencing a culture of struggle with the cost of living crisis and ensuring equitable access to of school food can help address this challenge  **Political culture:** There is a culture influenced by politics regarding school food, where ‘nanny state’ and preservation of autonomy were discussed as important, as well as Jamie Oliver’s influence in improving school food standards. |  | **Empowerment, choice preservation and autonomy**: The empowerment of parents and pupils through paying for meals was raised, as was the preservation of choice and autonomy of pupils. Ownership of school meals is important and limiting the choice that those on FSM sometimes experience is negative.  **Stigma**: Stigma for parents and pupils was discussed at length, and policies should aim to reduce stigma for those on FSM. (Overlapped with other themes)  **Impact on teachers**: Teachers are being distracted from their role by supporting pupils for pastoral food/poverty related issues.  **School community and cohesion around mealtimes**: Concerns were raised about the social isolation of those who bring packed lunches for dietary or religious reasons, but the sense of community and cohesion that school meals provide is a key benefit of extending school meal provision.  **Food preferences**: Child food preferences tend towards ‘rubbish’ food and waste healthier food; food quality and meal type important for uptake. |
| **Ethical context** | **FSM eligibility and access to pupil premium funding**: Schools can access pupil premium funding —intended for disadvantaged students and used for various needs—through FSM eligibility, impact on wider school funding for deprived pupils through school food has ethical connotations for any policy changes.  **Cost of living crisis gives moral obligation to act**: Due to struggles nationally, more people are in need of FSM and making the expansion of this both an ethical and moral need.  **Stigma** of FSM is pervasive in society (Overlapped with other themes)  **Only primary schools**: An ethical issue of extending FSM in primary but still excluding secondary should be considered.  **Move to Universal Credit** more equitable because itincludes working people. | **Schools have discretion** to give a free meal, and this is written into the education act. | **FSM eligibility indicates wider need for support**: If families are registered for FSM, they also need a team of people around them for support in other areas related to deprivation.  **Preservation of liberty and autonomy**: Schools restricting what FSM pupils can eat is an ethical issue, and the universal provision of school meals protects from stigma.  **Food waste:** Food waste may increase through uptake increase, but food culture may improve through social cohesion. |
| **Legal Context** |  | **Schools have discretion** to give a free meal, and this is written into the education act. |  |
| **Political Context** | **Current advocacy and momentum towards universal school meals and the national living wage:** Advocacy efforts towards universal free school meals is building in the UK as is towards the national living wage.  **Political acceptability**: Policymakers face the challenge of prioritising "food-only" policies when other pressing needs are evident elsewhere too. But the ‘usual suspects’ will be critical, and the framing of policy options are important particularly believability. Quality of school meals is sensitive too, as policy expansion may not increase nutritional content of meals (due to quality) as uptake increases. A 5-year time frame is important for framing advocacy efforts and evidence as it is the length of a political cycle. Celebrities have previously helped increase school food standards.  **Practical changes in administration:** In 2025 Universal Credit roll out and transitional protection ends and any increase in provision will require extra government funding. FSM eligibility is used to assess pupil premium funding, but this creates misleading statistics in politics. Reports will state ‘FSM increasing’ even though pupils who are no longer eligible remain on the register are not being removed.  **Complexity and inadequacy of benefits system:** The current benefits system is not fit for purpose, it is too complex to navigate, the current FSM allowance is not enough for schools, and FSM registration is used to access pupil premium funding. | **Local authority political aims and practicalities**: Regional government aims to embed health into all policies, they also have the ability to advocate to the government and need short- and long-term evidence for making policy decisions. Some LA staff don’t understand FSM enrolment processes themselves.  **Local authorities are enrolling pupils**: Some local authorities are enrolling pupils themselves into FSM and others make parents do it.  **Local authorities and schools financially struggling:** BCC bankruptcy and the financial position of other local authorities and schools remain challenging, also funding for any policy needs to come from government, and schools no longer make profit on catering.  **Champion within schools to lead catering key:** The role of a champion and advocate within schools to reform school meals is key. |  |
| **Epidemiological context** |  | **FSM eligibility indicates wider need for support:** If families are registered for FSM, they also need a team of people around them for support in other areas related to deprivation. | **Cost of living crisis**: The UK is experiencing a cost-of-living crisis which impacts not only food poverty but wider deprivation and associated outcomes. Food impacts on behaviour, teacher engagement, eating habits and debt accrued by families |
| **Geographical context** |  | **Logistics and delivery of meals is important:** There is variation in the delivery and contracting of catering between local authorities and schools |  |
| **Implementation process** | **Advocacy surrounding lived experience:** Key elements of successful advocacy are demonstrating the wider benefits to school and education, particularly of a universal offer, storytelling approaches and emphasis that an extension of FSM is morally the right thing to do.  **Clear demonstration of benefits and fairness in policy options**: It’s important for policymakers to know the benefits of what is being implemented. They also need focus on fairness, engagement and buy-in of all levels centred on why food should be a focus above other things. Clear evidence will help show the greatest impact for those in need to help combat ministers and civil servant views that there is not enough evidence. Evidence of implementation success is also important as well as outcomes.  **Other government advocacy strategies**: It is difficult not to highlight the quality of school meals being variable and advocacy should focus on ‘fed is best’ messaging. Local authorities can also be used to advocate upwards to the government. | **Variation between local authorities and schools in school meals practices:** Variation across how FSM eligibility is assessed (LA doing it or parents), how much schools charge for meals, how much FSM allowance goes on food vs other things, on the acceptability of transitional protection, variation between school structures in middle schools and academies. All these need to be considered when trying to implement policy nationally. However, adaptability of schools to new policy shouldn’t be underestimated given previous adaptations.  **School based policies**: Using FSM % would be better than deprivation score, particularly those that are decile based. School based policies exclude some pupils though and it would be challenging to identify schools, seems like an unkind policy. However, using 50% FSM threshold would probably have quite high acceptability.  **Advocacy to LAs and schools:** Showing educational impacts, reduced costs and demonstrate it being ‘worth’ money being spent only on food is key for advocacy to schools and LA. An in-house champion in schools is key to engagement however it is notoriously difficult to engage with schools. Schools want proof and clear instructions on how to implement.  **FSM extension combined with other interventions:** Increased uptake may increase food waste, any extension to FSM could be combined with waste reduction and food quality interventions because they are interconnected. Schools could also introduce debt reduction interventions to support families.  **Practical constraints surrounding assessment of eligibility on policy options except universal offer:** It is unsure how to know at LA and school level who is on Universal Credit, or level of earnings. It would be challenging to get this data or get parents to disclose their income. Communication of eligibility needs is improving though, however currently LAs have to explain to parents what FSM process is.  **Current meal quality and acceptability:** Meal quality and previous meal quality may impact acceptability of any policy. Parents believe £2.60 is quite a lot to pay already and a charitable donation may be more acceptable to parents than them subsidising FSM. | **Stigma reduction in policies**: Universal offer is the best policy to remove stigma, however rebound stigma in secondary school needs to be accounted for. Schools with higher FSM rates have less stigma and policies need to include working poverty.  **Challenges with acceptability:** Compulsory school lunches can work but may have acceptability issues, also difficulty in getting support for any school level policy creates postcode lottery. Individual choice and liberty must be maintained. There is need to be aware of stigma, particularly because those that are ‘newly’ eligible may have less resilience than those who have always been on FSM. The importance of ‘nobody missing out’ is very crucial and spending money only on food may be challenging.  **Parent subsidy policies specifically unsure of acceptability:** This option has less research surrounding it so more information and evidence are needed, however it could be more equitable and socially acceptable. Some believes it could cause more stigma but could also make people feel empowered. |
| **Implementation strategy** | **Systems approaches and positive impacts to areas other than just food should be demonstrated:** For policies to be adopted, advocacy efforts and selection is dependent on benefits demonstrated in other parts of the system, for example educational outcomes. Communication can build on other advocacy efforts e.g. national living wage, food quality standards but tailoring of findings to audience is important (95% confidence intervals not accessible to all).  **Nationwide implementation needs to provide guidance and systems to support schools:** Areas such as auto-enrolment, the current benefits system complexity could be improved to support school meals policy implementation. Policies that create a cliff edge should be avoided and are less desirable politically. The linking of pupil premium to FSM should be changed so FSM eligibility can actually be about food. | **Local authority decision making processes depending on cost and health outcomes:** Cost benefit analyses are a helpful tool for local authorities to make decisions, their decision making is for wider outcomes as well as health and nutrition. Assumptions are that nutritional quality will increase with uptake may not be evidence based because school food standards adherence isn’t monitored.  **Process of eligibility and registration for FSM needs to reduce barriers and burden on parents/families:** Any policy with an income threshold, or that involves registration needs to make sure they aren’t dependent on families identifying themselves. Policies can be implemented without this to eliminate stigma.  **Practical challenges for schools of any increase in uptake:** Uptake may increase costs to schools due to administrative burden, capacity in kitchens and in-space requirements, also a challenge will be scheduling lunchtimes to accommodate for increased uptake. Extra one-off funding may be needed to accommodate for these.  **Recommendations for implementation in schools:** Ensure variation between schools are accounted for, particularly the flat cost of meal production. Engage champions in schools and provide schools detailed instructions on how to implement school meals related policy changes. Chefs in schools have good advice and support. Any parent subsidy approach should communicate to parents clearly where the money goes. Schools could make some school meals compulsory for certain year groups or key stages to guarantee uptake numbers.  **Challenges specifically for policies that focus on ‘school based’ segmentation:** Schools fluctuate within FSM criteria and deprivation statistics, therefore implementation of any cut-off could be challenging. These policies with a postcode lottery could make some schools competitive to get into, and brutal cliff edge cut offs should be avoided. | **Social and acceptability impacts of school meals policies:** stigma reduction a priority and concerns are that stigma in secondary school is not impacted by primary only policies. Pupil preferences and understanding the tipping point from packed lunches to school meals are important to increase uptake, particularly important is making meals pupils like and involving them in the menu design process. A focus on making sure pupils have any food should be the goal initially with shifting to better quality nutritional food a second goal. Communication of policies and how much of the funds go towards food is important for parents.  **Using previously established methods to make implementation and administration easier for parents:** auto-enrolment into FSM, processes used for preschool meals could be applied in primary settings, meal deals can encourage adherence to nutritional standards, local authorities can register parents instead of them doing it themselves. Attempts to avoid families running up debt are also important through chasing them as soon as it starts and implementing payment plans |
| **Implementation agents** | - Central government - National level policymakers - Civil servants - Academics - Advocacy stakeholders | - School administrators - Local authority - Regional Mayors - School kitchens - Catering companies - School leadership decision makers - Local authority administrators | - Families - Pupils - Parents - Teachers |
| **Implementation outcomes** | - Short and long term - 1 year, 5 year (political cycle), 10 years - Revenue decrease nationally of increased - uptake - Nutritional impacts - Employment - Education - Lifetime NHS costs - Hunger reduction - Diet related disease - Diet quality - Food insecurity - Food literacy - Job creation - Feedback loops and systems - Inequalities - Reduced household expenditure | - Reach and efficiency of policies at reaching right - people - Surplus costs - Meal quality on school level - School level food revenue - School level food waste - School dining facilities - Worklessness - Regional impacts - Registrations for FSM between authorities - FSM recorded vs FSM eligibility - School level economies of scale - Adherence to school food standards - Changes in timetables - Utilisation of school facilities | - Pupil stigma - Parent stigma - Social and peer relationships - Stigma impacts - Isolation of those on packed - lunches - Individual diet quality - Individual nutrition - Individual food waste - Academic achievement - Attention - Happiness - Comfort - Family struggle - Food quality - Time pupils spend eating - Social cohesion - Social acceptability |
